# Engineering multi-layered tissue constructs using acoustic levitation

**DOI:** 10.1038/s41598-019-46201-z

**Published:** 2019-07-05

**Authors:** Angela Tait, Peter Glynne-Jones, Alison R. Hill, David E. Smart, Cornelia Blume, Bjorn Hammarstrom, Adam L. Fisher, Martin C. Grossel, Emily J. Swindle, Martyn Hill, Donna E. Davies

**Affiliations:** 1 Brooke Laboratories, Clinical and Experimental Sciences, Faculty of Medicine, University of Southampton, Southampton, UK; 2 Mechanical Engineering, Faculty of Engineering and Physical Sciences, University of Southampton, Southampton, UK; 3 Institute for Life Sciences, University of Southampton, Southampton, UK; 4 School of Chemistry, Faculty of Engineering and Physical Sciences, University of Southampton, Southampton, UK; 5 Southampton NIHR Respiratory Biomedical Research Centre, University Hospital Southampton, Southampton, UK

**Keywords:** Apicobasal polarity, Adherens junctions, Biomedical engineering

## Abstract

Engineering tissue structures that mimic those found *in vivo* remains a challenge for modern biology. We demonstrate a new technique for engineering composite structures of cells comprising layers of heterogeneous cell types. An acoustofluidic bioreactor is used to assemble epithelial cells into a sheet-like structure. On transferring these cell sheets to a confluent layer of fibroblasts, the epithelial cells cover the fibroblast surface by collective migration maintaining distinct epithelial and fibroblast cell layers. The collective behaviour of the epithelium is dependent on the formation of cell-cell junctions during levitation and contrasts with the behaviour of mono-dispersed epithelial cells where cell-matrix interactions dominate and hinder formation of discrete cell layers. The multilayered tissue model is shown to form a polarised epithelial barrier and respond to apical challenge. The method is useful for engineering a wide range of layered tissue types and mechanistic studies on collective cell migration.

## Introduction

Epithelia line the outer surface of organs forming polarised structures that act as barriers to the external environment. In the conducting airways of the lungs, the bronchial epithelial surface protects against noxious gases, pathogens and particulates from the inhaled environment^1^. It is supported by a basement membrane consisting of the *lamina lucida* and *lamina densa*, which are composed of extracellular matrix (ECM) synthesized by the epithelium, and the *lamina reticularis* which is synthesised by resident fibroblasts. These stellate cells form an intermeshed layer within the basement membrane zone and cover about 70% of the *lamina reticularis*^2^. The close proximity of the fibroblasts to the epithelium facilitates intimate cell-cell communication and coordination of tissue responses by functioning as an epithelial-mesenchymal trophic unit (EMTU)^2,3^. In healthy individuals, the airway epithelium is approximately 35–45 µm thick and the basement membrane zone around 8 µm thick^4^. In asthma, there is an approximate doubling in the thickness of the basement membrane zone and dysregulation of the EMTU which contributes to disease pathogenesis^3,5,6^.

In chronic lung diseases like asthma, the failure to translate drug candidates from animal models to humans has led to a demand for more predictive models for mechanistic, pharmacological and toxicological studies^7^. This has yielded a range of co-culture models that provide an approximation of the EMTU, however they have several limitations including incorrect dimensionality. For example, epithelial cells can be grown on nanoporous membrane supports suspended above fibroblast cultures; in this case, the two cell types are many millimetres distant from each other and lack direct contact^8,9^. Although culturing epithelial cells and fibroblasts on the upper and lower surfaces of the nanoporous membranes brings the cell types into closer contact, they are still physically separated by a non-natural plastic structure that is *ca*. 10 µm thick, even though cell-cell contact may occur via the membrane pores^10,11^. Overlaying epithelial cells on fibroblasts embedded in collagen gels provides a more natural environment, however the gel is usually several millimetres thick, a gross exaggeration of the dimensions of basement membrane zone *in vivo*^12,13^.

An alternative method for creating direct contact co-culture models is through use of cell sheet engineering, a method developed to allow autologous cell sheets to be generated *in vitro* and then transplanted and engrafted *in vivo*^14^. This technique uses a thermoresponsive polymer such as Poly(N-isopropyl acrylamide) which is hydrophobic at 37 °C supporting cell attachment and growth to form a confluent sheet, but hydrophilic at temperatures below 32 °C enabling cell sheet release^15–17^. This method has been used to create co-cultures of keratinocytes and fibroblasts which remained as two distinct cell layers^18^. However, this approach involves physical manipulation of the cells both by exposure to low temperature for cell sheet release and by mechanical lifting to transfer the cell sheet which risks causing damage, especially if the cells are difficult to release from the substratum. In principle, a better approach to cell sheet engineering would be to create a monolayer of one cell type remote from any surfaces and to introduce that sheet to a layer of the second cell type, cultured traditionally on a substrate. This would offer the primary advantage of thermoresponsive polymers, without the need for exposure to low temperatures or mechanical damage.

Acoustic radiation forces can be used to manipulate cells remote from a substrate with minimal impact on cell viability^19,20^. Cells within an acoustic trap tend to be drawn into close proximity by lateral acoustic forces^21^ and by secondary Bjerknes forces^22^ enabling acoustic fields to be used to pattern cells on substrates^23,24^, to form multicellular agglomerates^25^ or spheroids^26^ and to study cell-cell interactions^27,28^. Here we report, for the first time, the use of acoustic radiation forces to facilitate production of a composite tissue structure by creating epithelial cell sheets that can be used to form a multi-layered structure that models the bronchial mucosa. We demonstrate that the behaviour of the epithelial cell sheets is critically dependent on the formation of adherens junctions in the acoustic bioreactor; this modifies epithelial cell behaviour so that they act collectively to form a sheet over a fibroblast layer. The resultant multi-layered construct is polarised and can respond to environmental stimuli. This cell sheet engineering approach using acoustic radiation forces is a useful tool for engineering composite cell structures to model multi-layered tissues *in vitro* and for mechanistic studies of collective cell migration.

## Results

### Monodisperse epithelial cells cannot form layered structures with fibroblasts

**Figure 1:**
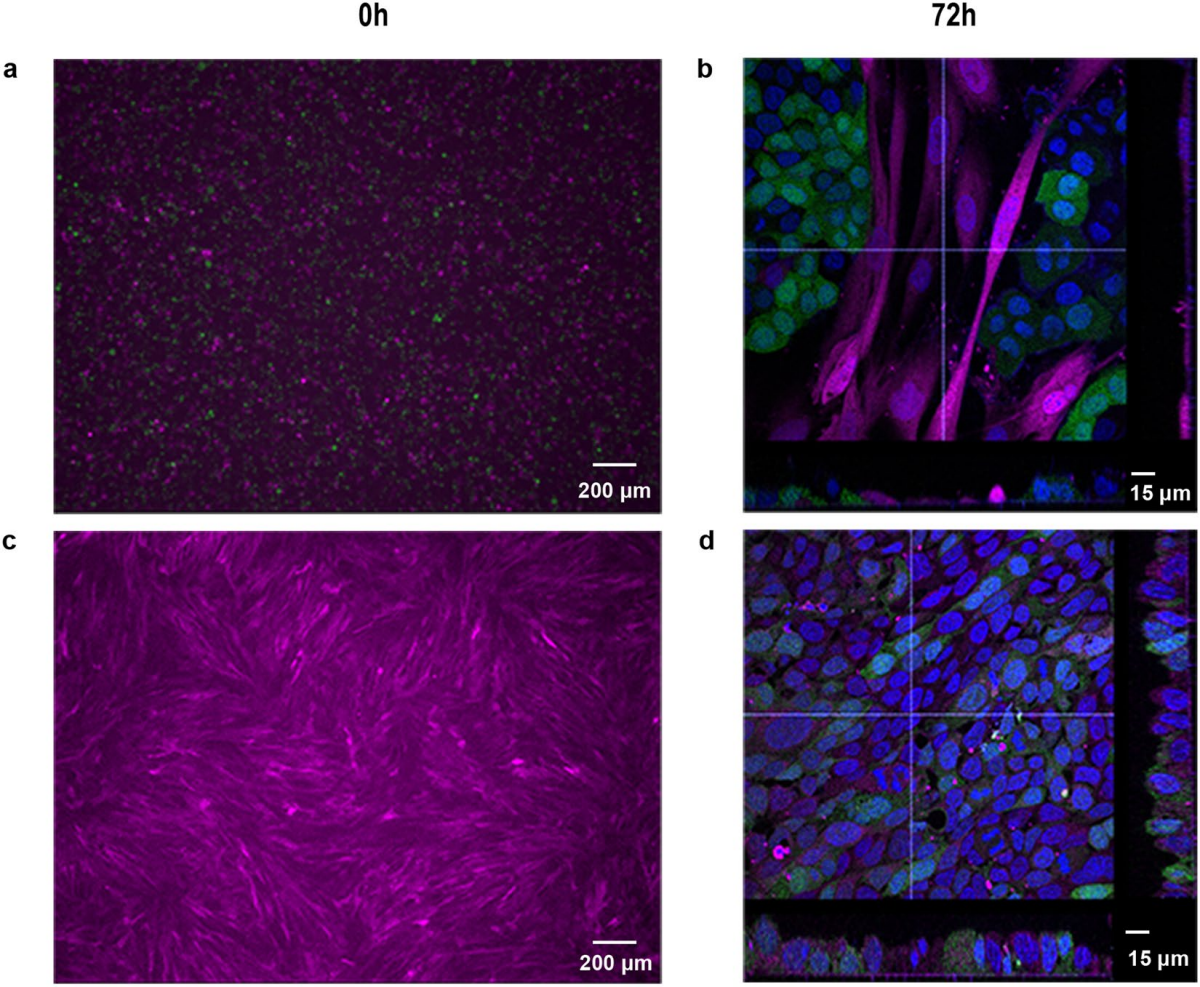
Co-culture of monodisperse epithelial cells with fibroblasts results in a random distribution of the two cell types. (**a**,**b**) A single cell suspension of epithelial cells (GFP-16HBE cells (green)) was mixed with a single cell suspension of fibroblasts (DsRed-MRC5 cells (magenta)); the images show the cells at 0 h (**a**) and after 72 h (**b**) of culture. (**c**,**d**) A confluent layer of DsRed MRC5 cells was established (**c**) prior to addition of a single cell suspension of GFP-16HBE cells and culture for 72 h. (**d**) Nuclei are labelled with DAPI (blue). Scale bar either 200 μm (**a**,**c**) or 15 μm (**b**,**d**).

Exploratory experiments revealed that application of monodisperse bronchial epithelial cells to fibroblast cultures resulted in formation of islands of epithelial cells surrounded by fibroblasts (Fig. 1a,b). This behaviour was attributed to the motile nature of the fibroblasts which allowed individual epithelial cells to make focal contacts with the substratum and establish strong adhesions followed by colony formation (Supplementary Video 1). Furthermore, bronchial epithelial sheets could not be released from thermoresponsive polymers (Supplementary Fig. 1) precluding this approach for cell sheet engineering. Thus, we hypothesised that formation of a multi-layered structure can be achieved using a bronchial epithelial sheet created remotely from a substrate using acoustic radiation forces.

### Epithelial cell behaviour in the acoustofluidic bioreactor

**Figure 2:**
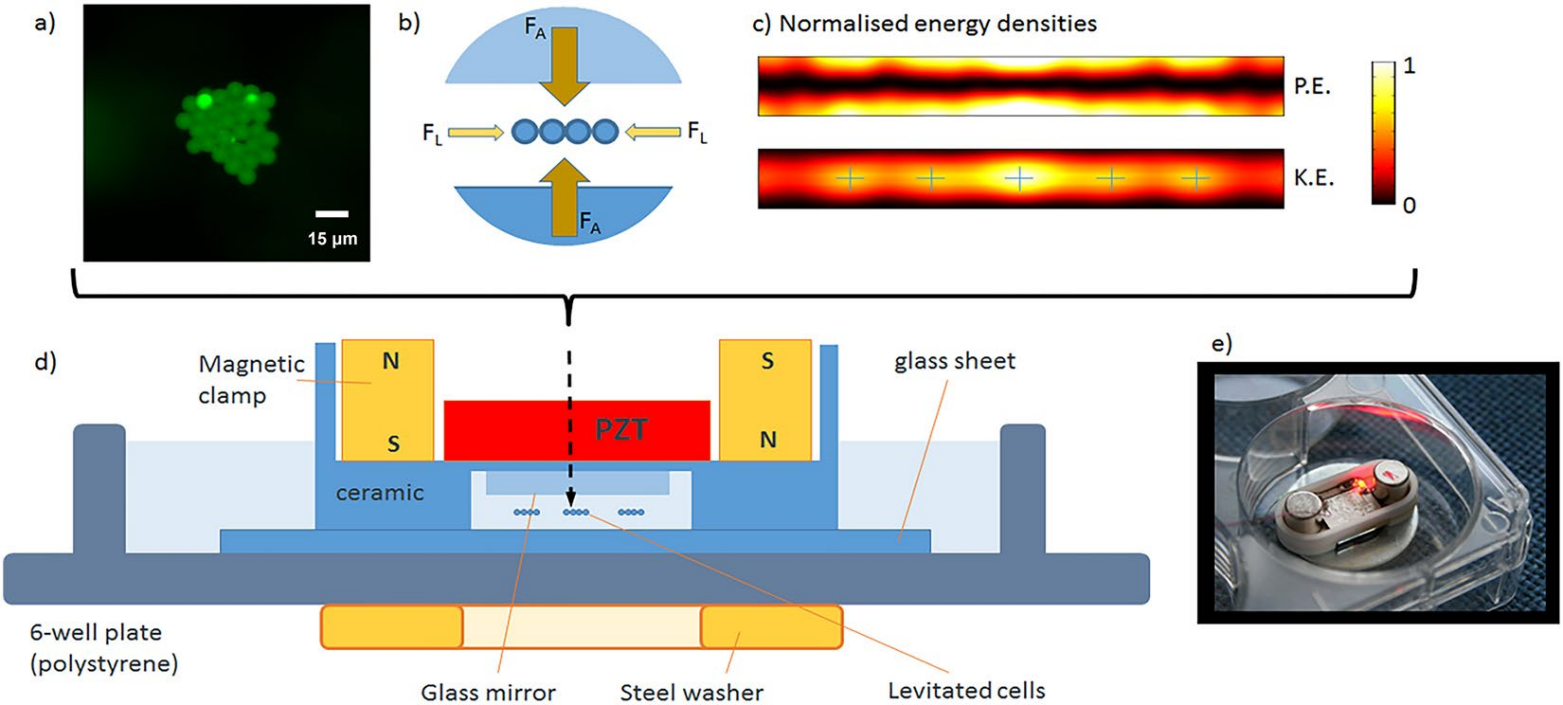
Design of the acoustic levitation device for preparation of epithelial cell sheets. The transducer creates an acoustic resonance in the medium-filled cavity beneath the mirror. Cells or microspheres are suspended in the centre plane of the cavity, scale bar is 15 μm and image taken by PGJ. (**a**) Acoustic forces are strongest in the axial direction (*F*_*A*_), with a weaker lateral component (*F*_*L*_) bringing cells together and forming an epithelial cell sheet. (**b**) Modelling of the acoustic field^21^, confirms that there is a pressure nodal plane (zero potential energy density – P.E.) corresponding to the levitation plane, within which, there are local maxima (marked with cross-hairs) in the kinetic energy density (K.E.) at which the mono-layer aggregates are formed. (**c**) Representative schematic diagram (**d**) and image of device (**e**). Not to scale.

The acoustic bioreactor design is shown in Fig. 2. The thicknesses of the layers within the structure were chosen using a transfer impedance model^29^ to create a strong half-wavelength acoustic resonance within a fluid-filled levitation chamber. Acoustic radiation forces resulting from sound scattered by particles/cells within the cavity cause them to be levitated in plane at the chamber half-height. 2D modelling using the finite element package, COMSOL^21^, revealed that the smaller lateral component of the acoustic radiation force forms a series of acoustic traps that cause particles to be drawn together into distinct monolayer aggregates within the levitation plane (Fig. 2a–c). The devices were driven from a single signal using a frequency sweep in the range 1.95 to 2.12 MHz (swept at a rate of 50 Hz). The sweep allows for device-to-device variation in resonance frequency and has the advantage of allowing for small changes in resonance frequency due to any physical changes such as medium composition or temperature.

**Figure 3:**
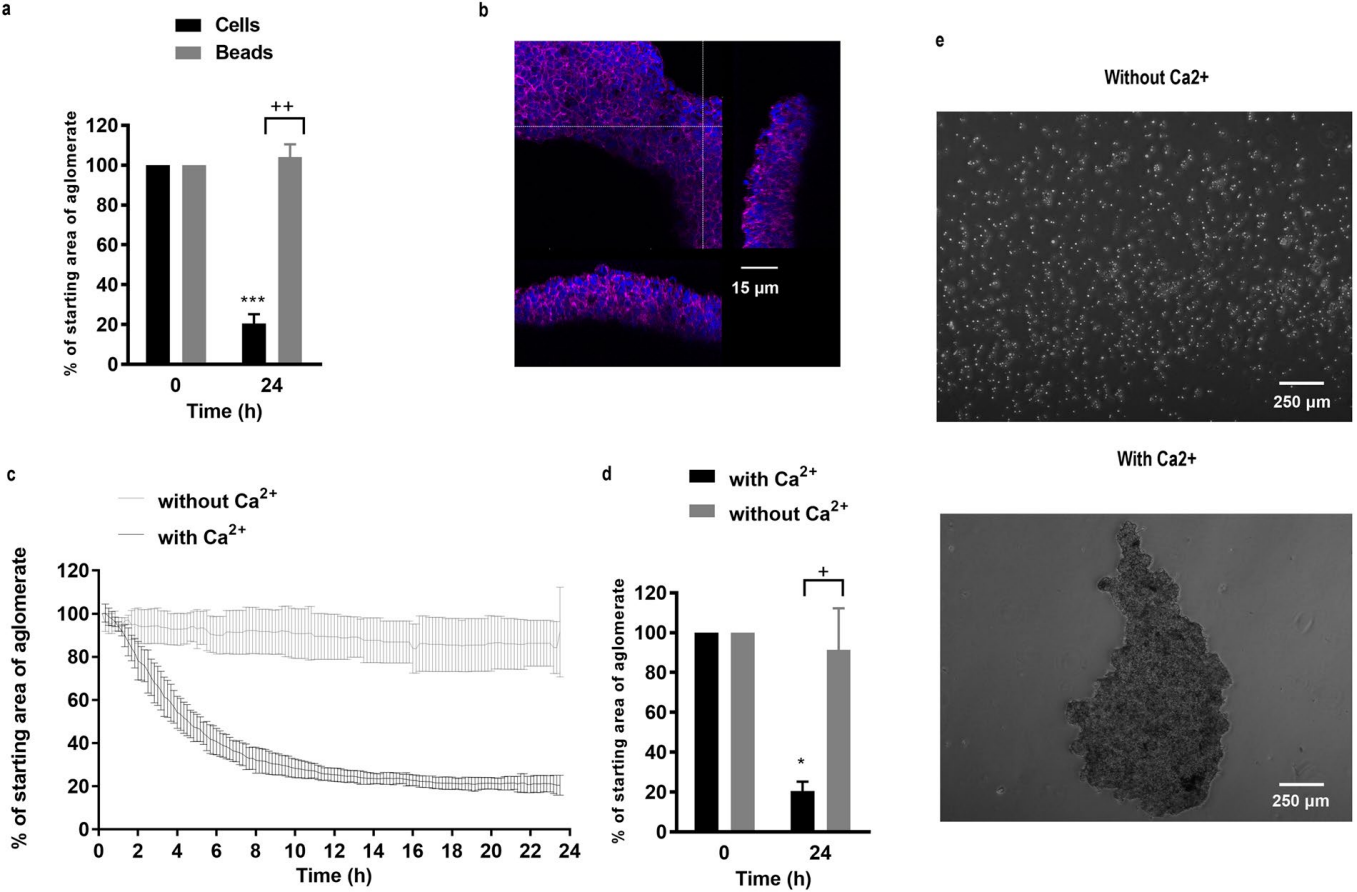
Epithelial cell behaviour in the acoustic bioreactor. Monodisperse suspensions of 10 µm polystyrene beads (**a**) or 16HBE cells (**a**–**e**) were introduced into the acoustic bioreactor and levitated for 24 h in the absence (c-e as indicated) or presence of Ca^2+^ (a–e as indicated); images were captured by time lapse microscopy and the resultant agglomerates were analysed and expressed as % of starting area (i.e. normalised to t = 0 h). Data are mean ± SD, n = 3 (**a**,**c**,**d**) or are representative images obtained by fluorescent microscopy for F-actin (magenta) and nuclei (blue) (**b**) or phase-contrast microscopy (**e**) (n = 3) of cells after levitation and removal from the bioreactor. Scale bars are 15 µm (**b**) and 250 µm (**e**) respectively. ***P ≤ 0.001 for comparison between 0 and 24 h and ^++^*P* ≤ 0.01 and ^+++^*P* ≤ 0.001 at 24 h compared to beads (**a**) or without calcium (**d**) (two-way ANOVA with Sidak’s correction for multiple comparisons).

Using these devices, suspended microspheres formed a monolayer whose area remained relatively stable over a 24 h period of levitation (Fig. 3a). Upon cessation of levitation, the beads returned to a monodisperse suspension. Similarly, epithelial cells could be levitated to form a monolayer in the acoustic bioreactor, however prolonged levitation resulted in reduction of the monolayer area due to formation of a 3D cellular agglomerate whose structure was stable after levitation (Fig. 3a,b, Supplementary Fig. 2). These data suggested that levitation and lateral corralling of the epithelial cells into a monolayer induced formation of adhesive complexes, followed by secondary contraction of the sheet to form a 3D spheroid.

**Figure 4:**
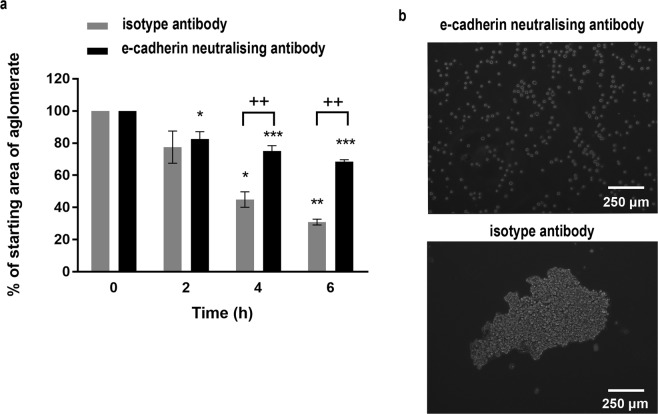
The effect of an E-cadherin neutralising antibody on formation of stable epithelial cell sheets. A single cell suspension of 16HBE cells was introduced into the acoustic bioreactor in the presence of a neutralising antibody to E-cadherin or isotype control and the size of the agglomerate area determined every 10 min for 6 h. Data are mean ± SD (**a**) or representative phase-contrast photomicrographs of the cell sheets after removal from the bioreactor following levitation for 6 h (**b**), n = 3. Scale bar is 250 μm. **P ≤ 0.01, ***P ≤ 0.001 compared to 0 h and ^+++^*P* ≤ 0.001 compared to E-cadherin neutralising antibody (two-way ANOVA with Sidak’s correction for multiple comparisons).

Epithelial adhesion depends on formation of adherens junctions involving the transmembrane protein, E-cadherin. This molecule mediates Ca^2+^-dependent homotypic adhesive interactions through its extracellular domain while the intracellular domain interacts with the actin cytoskeleton through the catenin family of adaptor proteins^30^. To determine whether adherens junctions were involved in stable cell sheet formation, we compared cell sheet formation without or with Ca^2+^ and the effect of a functional E-cadherin neutralising antibody. In the presence of Ca^2+^, the area of the levitated epithelial cells reduced to about 20–30% of the starting area within the first 6–8 h and then stayed relatively stable up to 24 h. In contrast, in the absence of Ca^2+^, cell monolayers formed but their areas were relatively constant during 24 h of levitation (Fig. 3c,d). After removal from the bioreactor, aggregates formed in the presence of Ca^2+^ were stable, whereas those formed in the absence of Ca^2+^ returned to a single cell suspension, reflecting the behaviour of microspheres (Fig. 3e). Similarly, cell monolayers were formed in the presence of a E-cadherin neutralising antibody but the reduction in area over 6 h was significantly less than for cells incubated with an isotype control antibody (Fig. 4a). Furthermore, after levitation for 6 h, the cell sheets formed in the presence of the E-cadherin neutralising antibody disassembled into single cells, whereas with the isotype control, the cells remained as an agglomerate (Fig. 4b). The requirement for both Ca^2+^ and E-cadherin for stable cell sheet formation identified involvement of adherens junctions in stable cell sheet formation.

**Figure 5:**
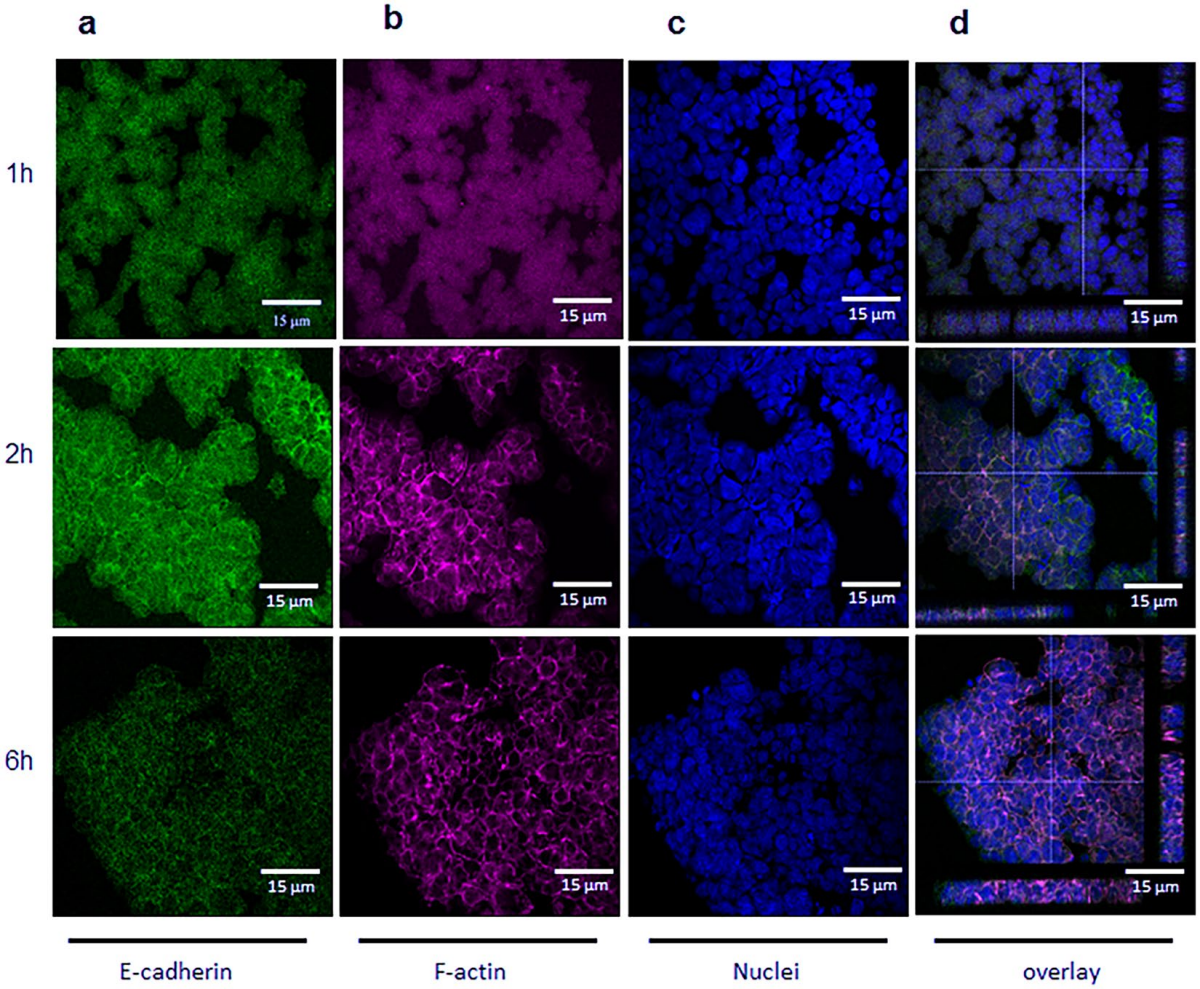
Formation of adherens junctions following levitation of epithelial cells. Representative fluorescent photomicrographs for E-cadherin (**a**, green), F-actin (**b**; magenta) or nuclei (**c**; blue) in epithelial cells after levitation within the bioreactor for 1, 2 or 6 h, as indicated (**a**–**c**); panel (d) shows a composite Z stack image for staining of a typical agglomerate at each time point. Scale bars are 15 µm and images are representative of 3 aggregates.

To optimise the time for cell sheet formation and confirm involvement of adherens junctions, epithelial cells were levitated for 1, 2 or 6 h and then fixed and stained for E-cadherin and F-actin (Fig. 5a–d). After 1 h of levitation, diffuse E-cadherin staining was observed with no obvious organisation of the actin cytoskeleton. After 2 h, E-cadherin and F-actin were localized at sites of cell-cell contact and the epithelial sheet comprised a single layer of cells. After 6 h, strong pericellular staining for both E-cadherin and F-actin was observed, however by this time the structure had become multi-layered (Fig. 5d). In all cases, the viability of levitated cells remained high (Supplementary Fig. 3). The time course for junctional assembly was similar to that seen in control epithelial cultures in which adherens junctions had been disassembled by removal of Ca^2+^ and then allowed to reform by reintroduction of Ca^2+^ (Supplementary Fig. 4). These data indicated that levitation of epithelial cells for 2 h allowed formation of a stable monolayer cell sheet mediated by adherens junctions.

**Figure 6:**
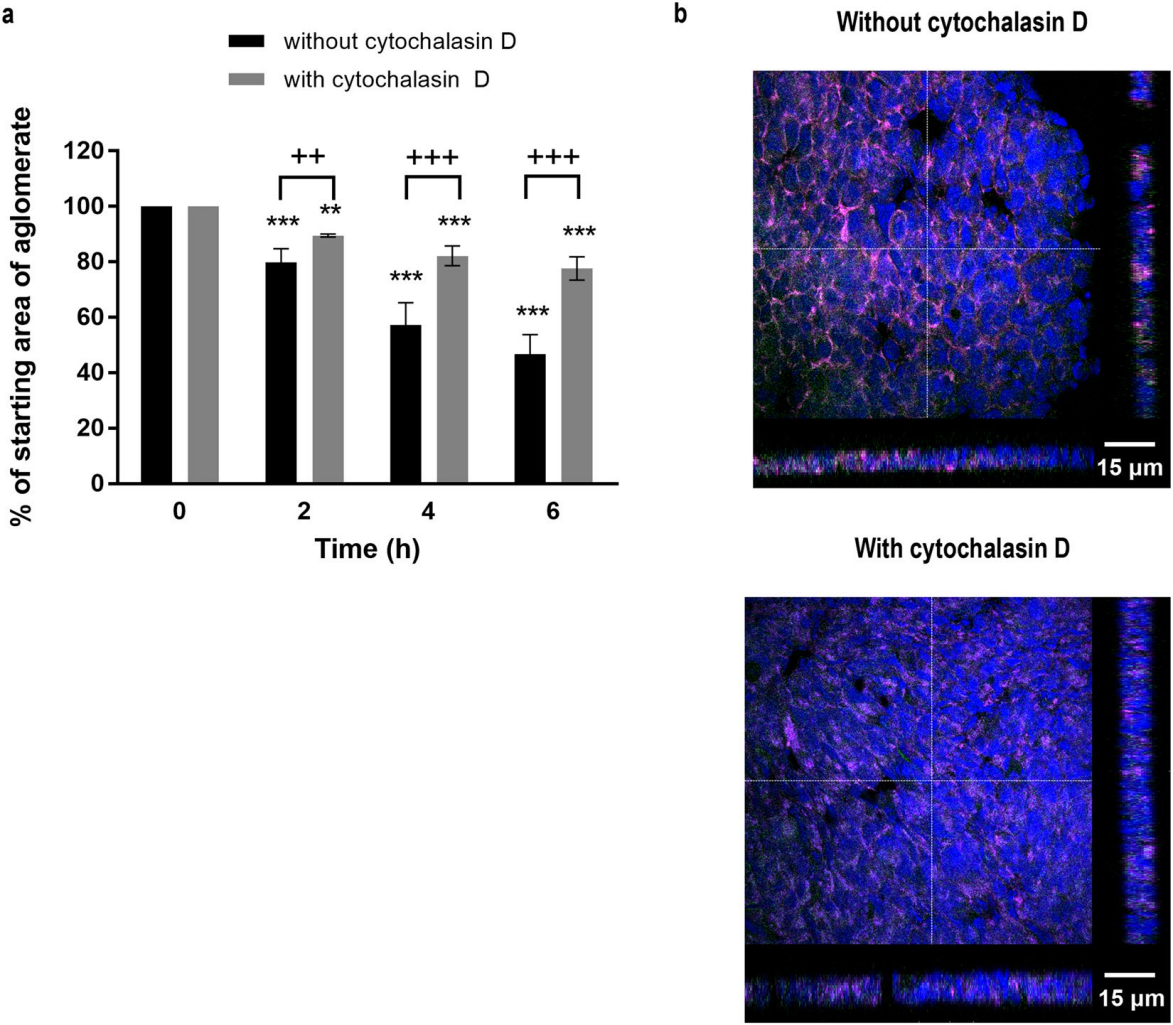
The effect of cytochalasin D on formation of epithelial cell sheets. A single cell suspension of 16HBE cells was introduced into the acoustic bioreactor in the absence or presence of cytochalasin D (2 μg/ml), and the size of the agglomerate area determined every 10 min for 6 h. (**a**) After 6 h, the cell sheets were removed from the acoustic bioreactor and E-cadherin (green), F-actin (magenta) or nuclei (blue) stained and visualised using fluorescent microscopy. (**b**) Data are mean ± SD (n = 4) (**a**) or representative Z stack images of aggregates obtained after levitation for 6 h in the absence or presence of cytochalasin D, as indicated (**b**), scale bar is 15 μm. **P ≤ 0.01, ***P ≤ 0.001 compared to control of t = 0 and ^+^*P* ≤ 0.05 and ^+++^*P* ≤ 0.001 compared to cytochalasin D (two-way ANOVA with Sidak’s correction for multiple comparisons).

To isolate the importance of the adherens junctions for formation of stable cell sheets from the unwanted contraction observed after cell sheet formation, we preincubated epithelial cells with cytochalasin D, an inhibitor of actin filament formation. This treatment significantly inhibited contraction of the levitated cell sheet (Fig. 6a) and confocal microscopy showed that whereas control cells had started to form multiple layers as a consequence of contraction, the cytochalasin D-treated cells maintained a monolayer structure after 6 h of levitation (Fig. 6b). Taken together, our data indicated that 2 h of levitation was sufficient for adherens junction formation and stabilisation of a monolayer cell sheet; levitation for longer provided no benefit and was associated with unwanted contraction of the epithelial cell sheets to form a 3D spheroid.

### Characterising multi-layered cellular constructs

**Figure 7:**
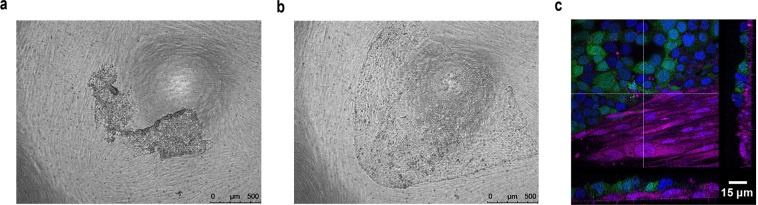
Formation of a multi-layered tissue construct with defined epithelial cell and fibroblast cell layers. A single cell suspension of GFP-16HBE cells was levitated for 2 h in the acoustic bioreactor to allow formation of an epithelial cell sheet with adherens junctions. The epithelial cell sheet was then removed from the bioreactor and placed on top of a confluent layer of DsRed-fibroblasts. (**a**) After 3 days of co-culture the epithelial cell sheet had grown as a sheet and not penetrated the fibroblast layer. (**b**,**c**) Results are representative phase-contrast photomicrographs at 0 h (**a**) or 72 h (**b**,**c**) where 16HBE cells (green), fibroblasts (magenta) or nuclei (blue) are visualised and displayed as a composite Z-stack image. (**c**) Scale bars are 500 μm (**a**,**b**) or 15 μm (**c**). n = 2.

To test the ability of the cell sheets to form co-cultures with fibroblasts, GFP-expressing bronchial epithelial cells were levitated for 2 h and the resultant cell sheets placed on top of a confluent layer of DsRed-expressing fibroblasts. Time-lapse microscopy over 3 days revealed that the cell sheet behaved in a co-ordinated manner, growing over the surface of the fibroblast layer by collective cell migration (Fig. 7a,b and Supplementary Video 2). Crucially, the epithelial cell sheet did not penetrate the fibroblast layer during the culture period resulting in defined layers of epithelial cells and fibroblasts (Fig. 7c). This was in contrast to the behaviour of control monodisperse epithelial cells in co-culture with a confluent fibroblast layer (Fig. 1d and Supplementary Video 3).

**Figure 8:**
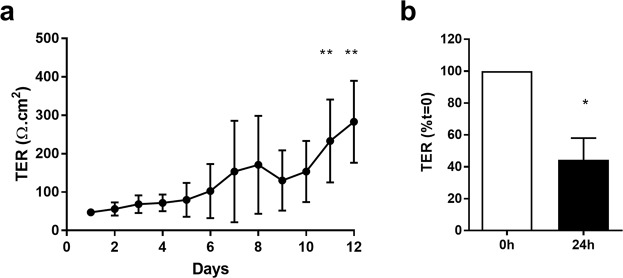
Functional analysis of the multi-layered airway construct. Establishment of an electrically tight epithelial sheet above the fibroblast layer was monitored daily by assessment of transepithelial electrical resistance (TER) (**a**); following formation of polarised tissue constructs (day 12), they were challenged with a viral mimetic, poly(I:C) (1 μg/ml) and effects on ionic permeability determined by monitoring TER. (**b**) Results are shown as means ± SD, n = 3–4 of TER readings expressed as Ω.cm^2^ (**a**) or % t = 0. (**b**) **P ≤ 0.01, compared to day 1 (Kruskal-Wallis with Dunn’s correction for multiple comparisons) and ^+^*P* ≤ 0.05 compared to t = 0 (paired two-tailed Student’s t-test).

Consistent with the fact that a continuous epithelial sheet had formed over the fibroblasts, the tissue construct developed an electrically tight barrier, as determined by transepithelial electrical resistance (TER) measurements which exceeded more than 250 Ω.cm^2^ (Fig. 8a). Furthermore, when challenged apically with dsRNA, an intermediate of replication of many common respiratory viruses and a danger signal recognised by pattern recognition receptors in epithelial cells, a significant decrease in ionic permeability was observed, (Fig. 8b) similar to that reported in other epithelial mono- and co-cultures^11,31^. These data illustrate the functionality of the multi-layered tissue construct.

## Discussion

We present a reusable acoustic device that can be mounted using a magnetic clamp inside a 6-well culture plate. Its layered design allows for a MHz standing wave resonance in the high aspect ratio manipulation chamber. The resulting acoustic radiation forces both levitate and bring together monodisperse epithelial cells within the levitation plane to form a coherent monolayer. Alignment of the device is made less critical by the use of a frequency sweep that allows for small deviations in the tuning of the acoustic resonance. By bringing the cells into intimate contact with each other, acoustic levitation facilitates formation of an interconnected cell sheet linked by adherens junctions. A key feature of this system is that cohesive cell sheets are formed rapidly ensuring that cell viability is not impaired despite lack of a substratum for anchorage. Furthermore, the adherens junctions mechanically stabilise the sheet, allowing its extraction from the acoustic trap and they subsequently play a crucial role in co-ordinating collective cell migration over a fibroblast layer to enable formation of two discrete cell layers that are functionally responsive. This model bears a closer spatial resemblance to airway tissue than the mainstay alternatives in which the cell layers are separated by plastic membranes or thick gels^32,33^.

Epithelial cells and fibroblasts grow *in vitro* in an ‘anchorage’-dependent manner which requires cell-to-matrix adhesion involving members of the integrin family^34^. In normal cells, loss of signals arising from these interactions usually results in programmed cell death^35^. Although cells are ‘anchored’ to the substratum, this does not prevent their movement via co-ordinated assembly and disassembly of integrin-mediated focal adhesions and reorganisation of the actin cytoskeleton. These dynamic processes play a role in both directed cell migration and random cell movement^36^. In our exploratory experiments, random movement of fibroblasts precluded generation of a multi-layered construct when monodisperse epithelial cells were placed on top of them, as the epithelial cells were able access the substratum and make their own focal adhesions. Thus, in this simple system, cell-matrix interactions dominated, and the behaviour of the epithelial cells as individual units prevented formation of distinct cell layers.

*In vivo*, *en masse* cell migration plays a crucial role in physiological processes of tissue formation, such as embryogenesis, morphogenesis, and wound healing^37^. In these situations, cells are influenced by the proximity of other cells as well as by ECM interactions and substrate mechanics. This collective cell behaviour can also be observed *in vitro*, for example in healing of scrape-wounded epithelial monolayers^38^. In response to wounding, a moving cell front is created allowing the epithelial cells to collectively migrate across the damaged area with a motion known as plithotaxis^34^. Such responses are controlled by co-operative intercellular forces involving transmission of appreciable stress across cell-cell junctions between neighbouring cells; after scrape wounding, the cells respond to changes in these forces by migrating along orientations of minimal intercellular shear stress at the wound edge^35^. By enabling formation of cell-cell adherens junctions before introducing cell-matrix interactions, acoustic levitation allowed us to change epithelial cell behaviour from that of the individual cell to that of the collective and to exploit the plithotactic response to achieve multi-layered tissue formation.

Acoustic fields have been used to pattern cells on substrates^23,24^, form multicellular agglomerates/spheroids^25,26^ and control intercellular communication^27,28^. Some of the ultrasonic fields used in such studies are excited by bulk acoustic waves^23,25,28^, while others employ surface acoustic wave excitation^24,26,27^ which is particularly suited to high-precision manipulation. The formation of cell sheets requires the levitation of cells to a single two-dimensional plane and this can be most easily achieved using a bulk acoustic wave planar resonant device in which the acoustic field is dominated by a one-dimensional axial variation of acoustic pressure^29^. Within such a field, cells are driven initially to the pressure nodal plane at the centre of a half-wavelength cavity, before being drawn together in local regions of high acoustic kinetic energy^21^. Acoustic radiation forces have been used to form levitated cell monolayers^22^ and to compare the adhesiveness of prostate epithelial cells with cancer cells^39^. Similar to our own findings, adherens junction formation was observed using prostate epithelial cells, however the prostate cancer cells showed reduced adhesiveness.

We demonstrated that both E-cadherin and Ca^2+^ were essential for formation of stable cell sheets in the acoustic bioreactor. Hepatocyte spheroid formation using a rocker technique also reported a requirement for Ca^2+^ and noted that non-adherent single cells died within 24 h^40^. We also showed involvement of the actin cytoskeleton in cell sheet formation however, over time, the contractile forces created by the actin cytoskeleton caused the sheets to form 3D spheroids, similar to those reported using other acoustic levitation devices^22^. It is likely that cell sheet contraction and spheroid formation reflects the long-term absence of a substratum for the cells to adhere to, as integrin-mediated cell-matrix interactions would normally oppose the contractile forces of the actin cytoskeleton across cell-cell junctions. By optimising the duration of levitation, we found that 2 h was sufficient for formation of stable monolayer sheets of cells with mature junctional complexes capable of directing collective cell behaviour when applied to fibroblast cultures. The resultant construct was functional as demonstrated by formation of a polarised electrically tight epithelial barrier^41^ and responsive as shown by the increase barrier permeability following challenge with dsRNA, a pathogen-associated molecular pattern, as observed in other epithelial systems^42^.

Epithelial-mesenchymal signalling plays a key role in tissue homeostasis and there has been a drive to develop more innovative, integrated models using, for example, nanoporous membranes, collagen gels or electrospun polymers to support the two cell types^7^. The advantage of the current model is that there is no need for artificial supports; this allows intimate contact between the epithelial cells and fibroblasts and the distances between the cell layers more closely mimic those found *in vivo*. Although cell sheets can be formed using thermoresponsive polymers, this approach it is not suitable for strongly adhesive cells. Here, we have used bronchial cells to demonstrate the feasibility of cell sheet engineering remote from a substrate using an acoustic bioreactor. This approach is equally applicable to other layered tissue models, such as skin, gut, vessels, and retina. Furthermore, we envisage the possibility of extending the method beyond two layered structures to create more complex structures. Beyond applications in tissue engineering, the acoustic bioreactor offers a new tool for mechanistic studies of collective cell migration, especially for studying cell-cell junctional stresses in the absence or presence of cell-matrix adhesion.

## Methods

### Cell culture

All cell culture products were obtained from Thermo Fisher Scientific (Inchinnan, UK) unless otherwise specified. The epithelial cell line 16HBE14o- (referred to as 16HBE, a gift from Professor D.C. Gruenert, San Fransisco, USA^43^ (passage 48–60) was maintained in Minimum Essential Medium (MEM) plus Glutamax^TM^ supplemented with 10% heat-inactivated foetal bovine serum (FBS) and penicillin (50 IU/ml)/streptomycin (20 μg/ml) (16HBE medium). The fibroblast cell line MRC5 (passage 40–50) was maintained in Dulbecco’s Modified Eagle’s Medium (DMEM) supplemented with 10% FBS and penicillin (50 IU/ml)/streptomycin (20 μg/ml), l-glutamine (2 mM), sodium pyruvate (1 mM), and non-essential amino acids (1 mM) (MRC5 medium). All cell lines were continuously cultured at 37 °C in 5% CO_2_ in air.

### Fluorescent cell lines

In some experiments, 16HBE cells were stained with CellTracker™ blue, according to manufacturer’s instructions. For co-cultures experiments, 16HBE cells were transfected with a plasmid encoding green fluorescent protein (GFP) under the control of a CMV promoter (pcDNA3.1, Clontech, California, USA) using TransIT-2020 transfection reagent (Mirus Bio, Madison, USA). MRC5 cells were transfected with pcDNA3.1 encoding red fluorescent protein (DsRed) by electroporation (Gene Pulser BioRad, Hemel Hempstead, UK). After 2 days (16HBE) or 3 days (MRC5) G418 (600 μg/ml) was used to select GFP- or DsRed-expressing cells and strongly expressing stable transfectants obtained by FACS sorting. These were maintained in medium containing 200 μg/ml G418.

### Acoustofluidic bioreactor fabrication

The acoustic levitation device, shown in Fig. 2, consisted of a unit that was magnetically clamped to a glass plate cut from a microscope slide. The resonant cavity was 0.38 mm in height and bounded by the device and the glass slide. The coupling layer of the device comprised a silvered mirror (glass face towards the chamber, measured as 0.9 mm thick) in order to create a dark-field background during fluorescent imaging. The body of the device was created by micro-milling of Macor^®^, a machinable ceramic. The mirror (Edmund Optics NT31-418, 9.5 mm × 11.2 mm) was attached to the Macor^®^ with an epoxy adhesive (Epo-tek^®^ 301) and cured at a temperature of 120 °C for 1 h. A piezo-electric material (lead zirconate titanate, PZT) transducer (PZ26, Ferroperm, Kvistgaard, Denmark, 1 mm × 10 mm × 11 mm) was attached to the Macor^®^ base using the same adhesive. The transducer electrodes were connected via soldered wires, with silver paint used to make connection between the lower electrode and a small area of the top electrode isolated by micromilling. A surface mount LED (Stanley, #UR1111C-TR, RS components, UK) with current limiting resistor was also attached to the transducer to indicate that power was reaching the transducer.

The assembled device was mounted within a 6-well microplate on top of a 1.2 mm thick microscope glass slide to act as an acoustic reflector. An M12 steel washer placed under the 6-well plate attracted magnets which were mounted in the device, thus holding it in position. This clamping was important as small movements of the device would disrupt the acoustic resonance (Fig. 2). A custom-made amplifier was used to drive the devices at an amplitude of 4 Vpp.

The acoustic pressure amplitude inside the capillary for a given drive voltage was estimated by balancing the weight of a 10 µm fluorescent polystyrene bead against the acoustic radiation force in the manner described by Spengler *et al*.^44^. Acoustic pressure was found to be related to drive voltage applied to the PZT by a factor of 26 kPa/Vpp ± 30%. The low accuracy of this measurement is caused by the difficulty of ascertaining when the two forces are precisely balanced, and uncertainty in the material properties of the polystyrene beads.

### Levitation of cells in the acoustofluidic bioreactor

Sterilised pre-cut transparent glass slides (2 cm^2^, Life Technologies) were attached to wells of a 6-well cell culture plate using silicon grease and cell culture medium added (3 ml). Sterilised acoustofluidic bioreactors were then placed on top of the glass slides inside each well, secured with a washer and the connectors attached. The pre-assembled bioreactors were transferred to a time-lapse microscope (LSM6000, Leica Microsystems, Wetzler, Germany) with a heated chamber at 37 °C, connected to a signal generator and power supply. After equilibration, a monodisperse suspension of fluorescent Fluoresbrite™ yellow green microspheres (10 µm; Polysciences Inc, Pennsylvania, USA) or 16HBE cells (5 × 10^3^ cells) was introduced into the bioreactor and the particles levitated for the times indicated in the figure legends. Images were recorded every 10 minutes using a time-lapse microscope (LSM6000, Leica Microsystems). In some experiments, the calcium chelator EGTA (5 mM, Sigma-Aldrich), cytochalasin D (2 µg/ml) or an E-cadherin neutralising antibody (1.6 µg/ml, clone SHE78-7, subclass mIgG2a) were added to the levitation chamber. Before the final image was taken, 7-Aminoactinomycin D (7-AAD; 10 μg/ml; Sigma-Aldrich, Poole, UK) was added to some bioreactors for 10 minutes to determine cell viability. At the end of the levitation period, cell sheets were allowed to settle to the bottom of the bioreactor and then transferred to poly-l-lysine-coated microscope glass slides (for immunofluorescent staining) or Transwell^®^ cell culture inserts (for co-culture) under sterile conditions. The time lapse images were used to calculate the area of the aggregated cells using ImageJ (National Institutes of Health, Bethesda, USA) with the Bio-Formats plugin.

### Immunofluorescent staining of cell sheets

Cell sheets on poly-l-lysine-coated microscope glass slides were washed in PBS, fixed with acetone: methanol (1:1, v/v) then permeabilized with Triton-X-100 (0.1% in PBS) before blocking with 1% BSA, 0.1% Tween 20 in PBS. Actin filaments were stained overnight at 4 °C with Acti-Stain™555-phalloidin or Acti-Stain™670-phalloidin, (Cytoskeleton, Denver, USA) and adherens junctions stained using anti-human E-cadherin antibody (clone HECD-1, subclass mIgG1k) conjugated to AlexaFluor^®^488 (using the Lightening-Link^®^ conjugation system (Innova Bioscience, Cambridge, UK) according to the manufacturer’s instructions). Nuclei were stained with DAPI (diamindino-2-pheylindole) and the slides mounted using ProLong^®^ Gold antifade reagent. Fluorescent images were acquired using either a LSM6000 fluorescent microscope (Leica) equipped with a 647/Cy5 filter cube (excitation 645 nm, emission 660 nm), a DAPI filter cube (excitation 365 nm, emission 440 nm) and a GFP/FITC filter cube (excitation 485 nm, emission 530 nm) prior to performing deconvolution (Leica Application Suite software). Or fluorescent confocal images were acquired using a laser scanning confocal microscope system (Leica TCS-SP5, Leica) equipped with a filterless tunable spectral detection system and attached to a Leica DMI6000 inverted microscope with or without sequential imaging, as required, to avoid spectral bleed through as follows for DAPI (excitation 405 nm, emission window 419–486 nm), GFP (excitation 488 nm, emission window 498–527 nm), DsRed (excitation 561 nm, emission window 569–620 nm), AF488 (excitation 488 nm, emission window 501–554 nm) and AF647 (excitation 663 nm, emission window 641–709) and displayed with pseudo-colouring.

### Co-culture model

MRC5 cells expressing DsRed (3 × 10^4^/well) were seeded into the apical compartment of Transwell^®^ cell culture inserts and cultured until confluent. 16HBE cell sheets obtained from the acoustofluidic bioreactor were transferred onto confluent MRC5 monolayers and the co-culture was incubated at 37 °C for up to 14 days. Cell behaviour was observed using time-lapse microscopy. Once established, co-cultures were challenged apically with 1 µg/ml dsRNA (polyinosinic:polycytidylic acid; poly(I:C) (Invivogen, San Diego, USA) to mimic a viral infection. Epithelial barrier function was monitored by measuring transepithelial resistance (TER) using chopstick electrodes connected to an EVOM Volt/Ohm meter (World Precision Instruments, Aston, UK) and expressed as ohms.cm^2^ (Supplementary Fig. 5).

### Statistical analysis

Data were tested for normality using the Shapiro-Wilk test. Parametric data were expressed as means ± standard deviation (SD). For parametric data, differences between 2 groups were tested for statistical significance using Student’s t-test while differences between >2 groups was determined by two-way ANOVA with Sidak’s correction for multiple comparisons. For non-parametric data, differences between groups was determined by Kruskal-Wallis with Dunn’s correction for multiple comparisons. All data were analysed using Prism (GraphPad Software, San Diego, USA). *P* < 0.05 were considered statistically significant.

## Supplementary information

Supplementary information

Related Manuscript File

Related Manuscript File

Related Manuscript File

## Data Availability

Data supporting this study are openly available from the University of Southampton repository (10.5258/SOTON/D0994).

## References

[1] Marchiando AM, Graham WV, Turner JR (2010). Epithelial barriers in homeostasis and disease. Annu Rev Pathol.

[2] Evans MJ, Van Winkle LS, Fanucchi MV, Plopper CG (1999). The attenuated fibroblast sheath of the respiratory tract epithelial-mesenchymal trophic unit. Am J Respir Cell Mol Biol.

[3] Davies DE (2009). The role of the epithelium in airway remodeling in asthma. Proc Am Thorac Soc.

[4] Jeffery PK, Wardlaw AJ, Nelson FC, Collins JV, Kay AB (1989). Bronchial biopsies in asthma. An ultrastructural, quantitative study and correlation with hyperreactivity. Am Rev Respir Dis.

[5] Xiao C (2011). Defective epithelial barrier function in asthma. J Allergy Clin Immun.

[6] Loxham M, Davies DE, Blume C (2014). Epithelial function and dysfunction in asthma. Clin Exp Allergy.

[7] Blume C, Davies DE (2013). *In vitro* and *ex vivo* models of human asthma. Eur J Pharm Biopharm.

[8] Swartz MA, Tschumperlin DJ, Kamm RD, Drazen JM (2001). Mechanical stress is communicated between different cell types to elicit matrix remodeling. Proc Natl Acad Sci USA.

[9] Reeves SR (2014). Asthmatic airway epithelial cells differentially regulate fibroblast expression of extracellular matrix components. J Allergy Clin Immunol.

[10] Pohl C (2009). Acute morphological and toxicological effects in a human bronchial coculture model after sulfur mustard exposure. Toxicol Sci.

[11] Hill AR (2016). IL-1alpha mediates cellular cross-talk in the airway epithelial mesenchymal trophic unit. Tissue Barriers.

[12] Choe MM, Sporn PH, Swartz MA (2003). An *in vitro* airway wall model of remodeling. Am J Physiol Lung Cell Mol Physiol.

[13] Paquette JS, Moulin V, Tremblay P, Bernier V, Boutet M, Laviolette M, Auger FA, Boulet LP, Goulet F (2004). Tissue-engineered human asthmatic bronchial equivalents. European Cells and Materials.

[14] Matsuura K, Haraguchi Y, Shimizu T, Okano T (2013). Cell sheet transplantation for heart tissue repair. J Control Release.

[15] Kushida A, Yamato M, Isoi Y, Kikuchi A, Okano T (2005). A noninvasive transfer system for polarized renal tubule epithelial cell sheets using temperature-responsive culture dishes. European Cells and Materials.

[16] Kushida A, Yamato M, Kikuchi A, Okano T (2001). Two-dimensional manipulation of differentiated Madin-Darby canine kidney (MDCK) cell sheets: the noninvasive harvest from temperature-responsive culture dishes and transfer to other surfaces. J Biomed Mater Res.

[17] Kushida A (2000). Temperature-responsive culture dishes allow nonenzymatic harvest of differentiated Madin-Darby canine kidney (MDCK) cell sheets. J Biomed Mater Res.

[18] Cerqueira MT (2014). Cell sheet technology-driven re-epithelialization and neovascularization of skin wounds. Acta Biomater.

[19] Bazou D (2011). Gene Expression Analysis of Mouse Embryonic Stem Cells Following Levitation in an Ultrasound Standing Wave Trap. Ultrasound Med Biol.

[20] Wiklund M (2012). Acoustofluidics 12: Biocompatibility and cell viability in microfluidic acoustic resonators. Lab Chip.

[21] Glynne-Jones P (2012). Array Controlled Ultrasonic Manipulation of Particles in Planar Acoustic Resonator. IEEE Transactions on Ultrasonics, Ferroelectrics and Frequency Control.

[22] Bazou D (2006). Gap junctional intercellular communication and cytoskeletal organization in chondrocytes in suspension in an ultrasound trap. Mol Membr Biol.

[23] Bernassau AL, Gesellchen F, MacPherson PGA, Riehle M, Cumming DRS (2012). Direct patterning of mammalian cells in an ultrasonic heptagon stencil. Biomed Microdevices.

[24] Collins DJ (2015). Two-dimensional single-cell patterning with one cell per well driven by surface acoustic waves. Nat Commun.

[25] Li S (2014). Application of an acoustofluidic perfusion bioreactor for cartilage tissue engineering. Lab Chip.

[26] Chen Kejie, Wu Mengxi, Guo Feng, Li Peng, Chan Chung Yu, Mao Zhangming, Li Sixing, Ren Liqiang, Zhang Rui, Huang Tony Jun (2016). Rapid formation of size-controllable multicellular spheroids via 3D acoustic tweezers. Lab on a Chip.

[27] Guo F (2015). Controlling cell-cell interactions using surface acoustic waves. Proc Natl Acad Sci USA.

[28] Christakou AE (2013). Live cell imaging in a micro-array of acoustic traps facilitates quantification of natural killer cell heterogeneity. Integr Biol.

[29] Glynne-Jones P, Boltryk RJ, Hill M (2012). Acoustofluidics 9: Modelling and applications of planar resonant devices for acoustic particle manipulation. Lab Chip.

[30] Takeichi M (1991). Cadherin cell adhesion receptors as a morphogenetic regulator. Science.

[31] Blume C (2017). Cellular crosstalk between airway epithelial and endothelial cells regulates barrier functions during exposure to double-stranded RNA. Immun Inflamm Dis.

[32] Chowdhury F, Howat WJ, Phillips GJ, Lackie PM (2010). Interactions between endothelial cells and epithelial cells in a combined cell model of airway mucosa: effects on tight junction permeability. Exp Lung Res.

[33] Hoang ATN (2012). Dendritic cell functional properties in a three-dimensional tissue model of human lung mucosa. Am J Physiol Lung Cell Mol Physiol.

[34] Giancotti FG, Tarone G (2003). Positional control of cell fate through joint integrin/receptor protein kinase signaling. Annu Rev Cell Dev Biol.

[35] Paoli P, Giannoni E, Chiarugi P (2013). Anoikis molecular pathways and its role in cancer progression. Biochim Biophys Acta.

[36] Collins C, Nelson WJ (2015). Running with neighbors: coordinating cell migration and cell-cell adhesion. Curr Opin Cell Biol.

[37] Hunter MV, Fernandez-Gonzalez R (2017). Coordinating cell movements *in vivo*: junctional and cytoskeletal dynamics lead the way. Curr Opin Cell Biol.

[38] Molinie N, Gautreau A (2018). Directional Collective Migration in Wound Healing Assays. Methods Mol Biol.

[39] Bazou D, Davies G, Jiang WG, Coakley T (2006). Rapid molecular and morphological responses of prostate cell lines to cell-cell contact. Cell Commun Adhes.

[40] Brophy CM (2009). Rat Hepatocyte Spheroids Formed by Rocked Technique Maintain Differentiated Hepatocyte Gene Expression and Function. Hepatology.

[41] Wan H (2000). Tight junction properties of the immortalized human bronchial epithelial cell lines Calu-3 and 16HBE14o. Eur Respir J.

[42] Comstock AT (2011). Rhinovirus-induced barrier dysfunction in polarized airway epithelial cells is mediated by NADPH oxidase 1. J Virol.

[43] Cozens AL (1994). CFTR expression and chloride secretion in polarized immortal human bronchial epithelial cells. Am J Respir Cell Mol Biol.

[44] Spengler JF (2001). Observation of yeast cell movement and aggregation in a small-scale MHz-ultrasonic standing wave field. Bioseparation.

