# Supplementary material for: Engineering multi-layered tissue constructs using acoustic levitation
Source: Sci Rep. 2019 Jul 5;9:9789. doi: 10.1038/s41598-019-46201-z (PMC6611909; doi:10.1038/s41598-019-46201-z)
Supplement: Supplementary file 1 — Supplementary information [file 41598_2019_46201_MOESM1_ESM.pdf]

Engineering multi-layered tissue constructs using acoustic levitation.

Angela Tait[1+], Peter Glynne-Jones[2,3+], Alison R. Hill[1], David E. Smart[1], Cornelia Blume[1], Bjorn Hammarstrom[2], Adam L Fisher[4], Martin C. Grossel[3,4], Emily J. Swindle[1,3,5], Martyn Hill[2,3#], Donna E. Davies[1,3,5#*]

[1]Brooke Laboratories, Clinical and Experimental Sciences, Faculty of Medicine, [2]Mechanical Engineering, Faculty of Engineering and Physical Sciences, [3]Institute for Life Sciences, [4]School of Chemistry, Faculty of Engineering and Physical Sciences, University of Southampton, Southampton, UK, and the [5]Southampton NIHR Respiratory Biomedical Research Centre, University Hospital Southampton, Southampton UK.

[+]joint first authors, [#]joint last authors and *corresponding author

Address for correspondence:

Professor Donna E Davies

Clinical and Experimental Sciences,

Sir Henry Wellcome Laboratories

Faculty of Medicine, University of Southampton,

University Hospital Southampton,

Tremona Road, Southampton, SO16 6YD, UK.

Tel +44 (0)23 81208523

FAX +44 (0)23 80511761

Email: D.E.Davies@southampton.ac.uk

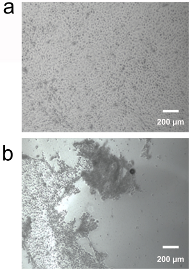

Supplementary Figure 1. Incomplete epithelial cell sheet removal using a thermoresponsive polymer. 16HBE cells were grown to confluency on a commercial thermoresponsive polymer (poly(N-isopropyl acrylamide) coated dish (UpcellTM) (a). After incubation with a membrane on top of the cell layer for 0.5 h at 20°C, the membrane was lifted with attached cells and the cells left attached to the dish were imaged using phase contrast microscopy (b). Scale bars are 200 µm.

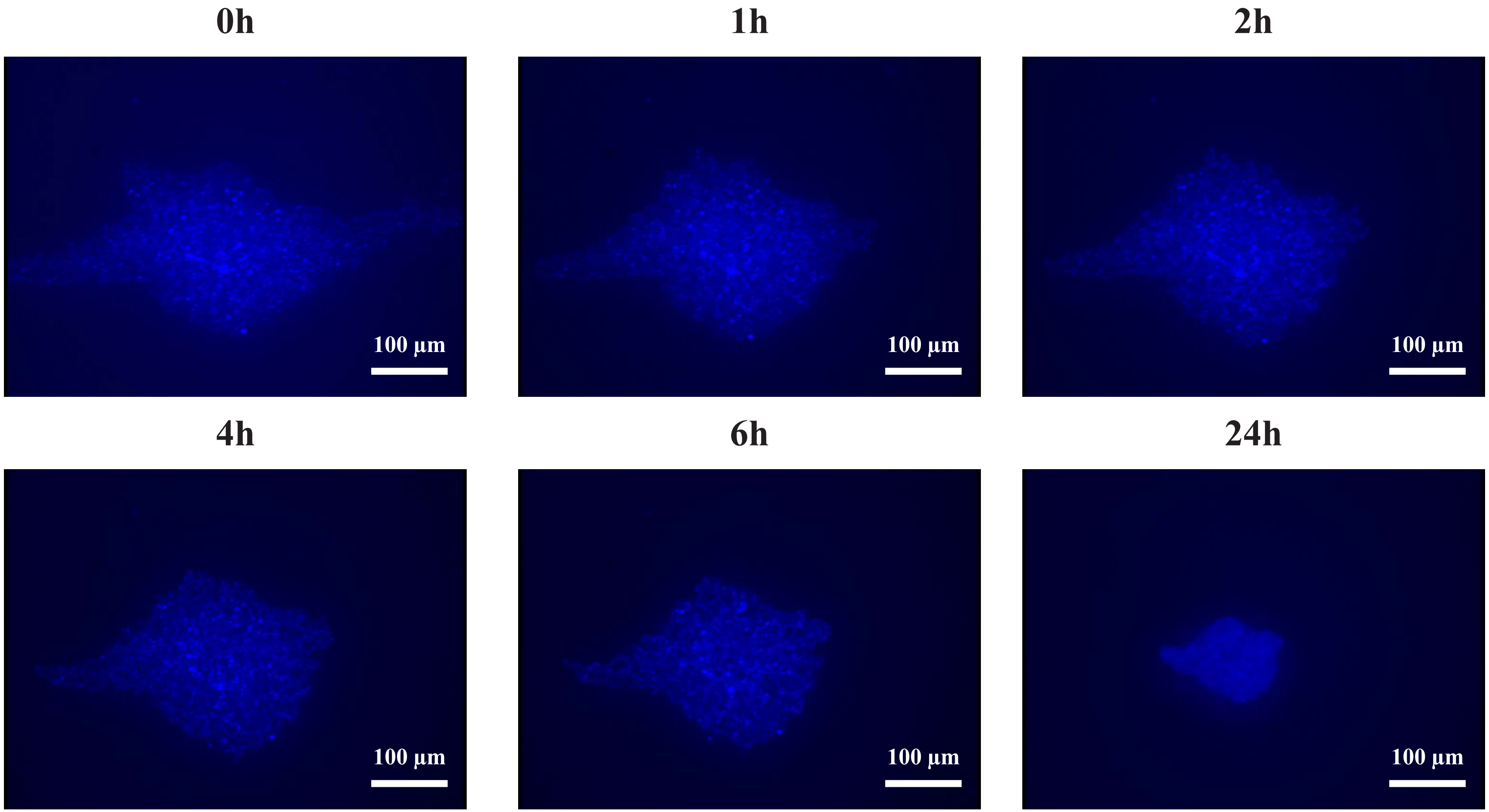

Supplementary Figure 2. Epithelial cell sheet size over time in the acoustic bioreactor. A single cell suspension of cell-tracker blue-stained 16HBE cells was introduced into the acoustic bioreactor and levitated for 0-24 h. Data are representative images captured by time lapse fluorescent microscopy at each time point. Scale bars are 100 µm.

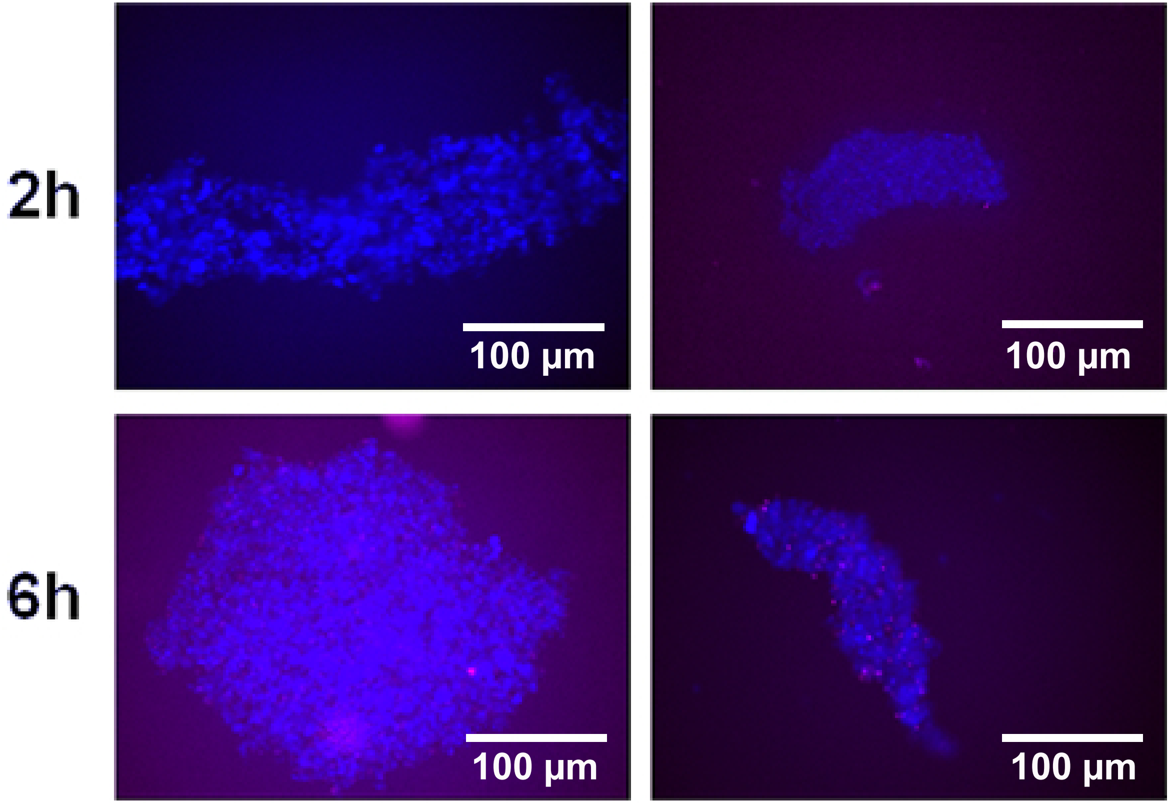

Supplementary Figure 3. Epithelial cell viability over time in the acoustic bioreactor. A single cell suspension of cell-tracker blue-stained 16HBE cells was introduced into the acoustic bioreactor and levitated for 2 or 6 h. Ten minutes prior to the end of levitation 7-AAD was introduced into the bioreactor to assess viability after 2 h (a) or 6 h (b). Data are representative images obtained by fluorescent microscopy. Scale bars are 100 μm.

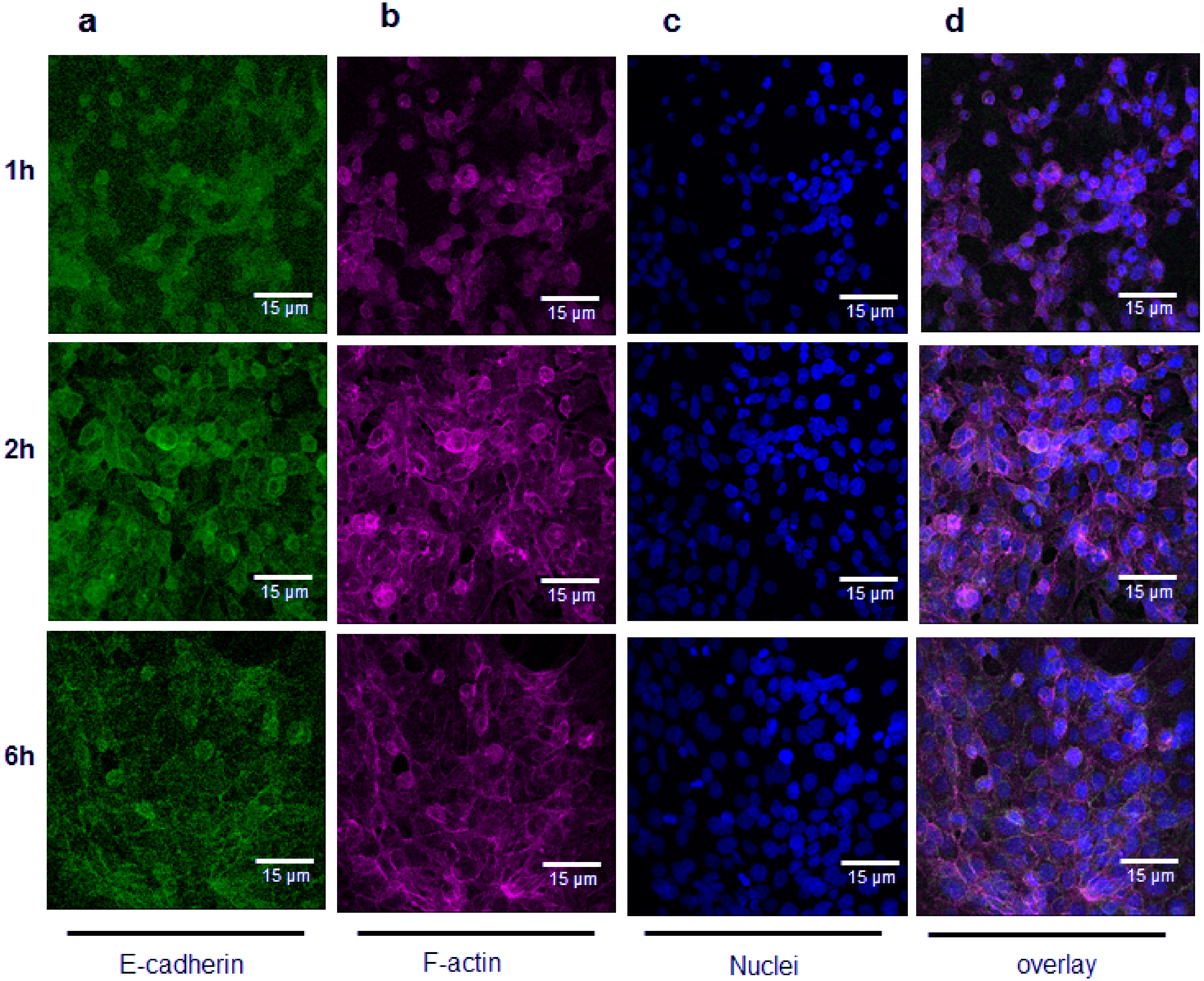

Supplementary Figure 4. Formation of adherens junctions in epithelial cell monolayer cultures. 16 HBE epithelial cells were grown to confluency on coverslips before Ca^{2+} was removed from the culture medium for 4 h to cause disassembly of adherens junctions. The medium was then resupplemented with Ca^{2+} and re-formation of adherens junctions followed at 1, 2 and 6 h. The figure shows representative fluorescent photomicrographs of E-cadherin (a, green), F-actin (b; magenta) or nuclei (c; blue) at each time point, as indicated; panel (d) shows a composite Z stack image for staining of a typical agglomerate. Scale bars are 15 μm.

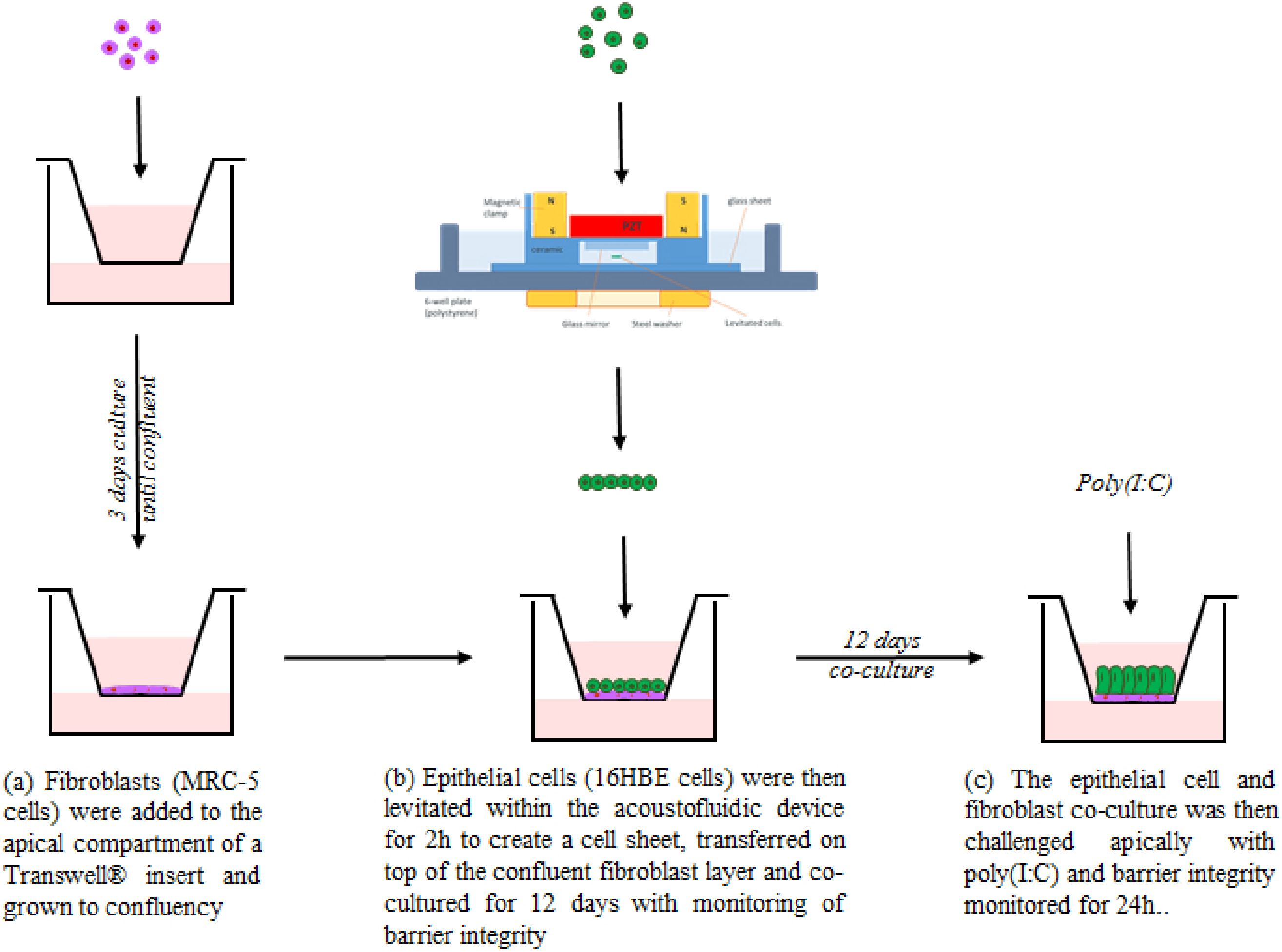

Supplementary Figure 5. Schematic of establishing the epithelial cell and fibroblast co-culture model. A confluent layer of DsRed MRC5 cells (magenta) was established (a) prior to addition of an epithelial cell sheet generated in the acoustic bioreactor (b) and co-cultured for 12 days with daily monitoring of barrier integrity by TER measurements. After 12 days, the co-culture was challenged with a viral mimetic, poly(I:C) (1 µg/ml) and effects on ionic permeability determined by monitoring TER (c).

Supplementary Video Captions

Supplementary Video 1. Co-culture of monodispersed epithelial cells and fibroblasts results in a random mixture of the two cell types. A single cell suspension of both DsRed MRC5 cells (red) and GFP-16HBE cells (green) were mixed together and cultured for 72 h. Cell behaviour over the 72 h was followed by time-lapse video microscopy.

Supplementary Video 2. Migration and growth of an epithelial cell sheet over a fibroblast layer demonstrates collective cell migration resulting in two discrete cell layers. A confluent layer of DsRed MRC5 cells (red) was established prior to addition of an epithelial cell sheet generated in the acoustic bioreactor and cultured for 72 h. Data are displayed as a time-lapse microscopy video of phase-contrast images over 72 h.

Supplementary Video 3. Migration and growth of monodispersed epithelial cells over a fibroblast layer results in undefined mixed cell layers. A confluent layer of DsRed MRC5 cells (red) was established prior to addition of a single cell suspension of 16 HBE epithelial cells and culture for 72 h. Cell behaviour over the 72 h was followed by time-lapse video microscopy.
